# Diabetic Microangiopathy Is an Independent Predictor of Incident Diabetic Foot Ulcer

**DOI:** 10.1155/2016/5938540

**Published:** 2016-02-29

**Authors:** Masuomi Tomita, Yusuke Kabeya, Mari Okisugi, Takeshi Katsuki, Yoichi Oikawa, Yoshihito Atsumi, Kempei Matsuoka, Akira Shimada

**Affiliations:** ^1^Department of Internal Medicine, Tokyo Saiseikai Central Hospital, Tokyo 108-0073, Japan; ^2^Division of General Internal Medicine, Department of Internal Medicine, Tokai University Hachioji Hospital, Tokyo, Japan; ^3^Diabetes Center, Eiju General Hospital, Tokyo, Japan; ^4^Saiseikai Shibuya Satellite Clinic, Tokyo, Japan

## Abstract

*Aim.* To determine the diabetic foot ulcer incidence and examine its association with microangiopathy complications, including diabetic retinopathy (DR) and albuminuria (Alb), in type 2 diabetes patients.* Methods.* This was a retrospective cohort study of 1,305 patients with type 2 diabetes who were assigned to the following groups: Category 1, normoalbuminuria without DR (*n* = 712); Category 2, Alb without DR (*n* = 195); Category 3, normoalbuminuria with DR (*n* = 185); and Category 4, Alb with DR (*n* = 213). Cox proportional hazard models were used to compare the risks of developing diabetic foot ulcers across the categories.* Results*. During 14,249 person-years of follow-up, 50 subjects developed diabetic foot ulcers, with incidence rates of 1.6/1,000, 1.5/1,000, 3.4/1,000, and 12.5/1,000 person-years in Categories 1, 2, 3, and 4, respectively. After adjusting for the presence of diabetic neuropathy and macroangiopathy, the hazard ratios and 95% confidence intervals (CIs) for the risk of diabetic foot ulcer development were 0.66 (95% CI, 0.18–2.36), 1.72 (95% CI, 0.67–4.42), and 3.17 (95% CI, 1.52–6.61) in Categories 2, 3, and 4, respectively, compared with Category 1.* Conclusion*. The presence of DR and Alb significantly increases the risk of diabetic foot ulcer development.

## 1. Introduction

Diabetic complications have become a serious issue in Japan, and the dramatic rise in the number of patients with diabetes has exacerbated this problem. However, awareness of one serious health condition, namely, diabetic foot disease, is inadequate among patients and healthcare providers [1, 2]. In 2008, the Japanese government introduced a management fee for diabetic complications to facilitate the prevention of diabetic foot ulcers. Consequently, the number of foot care outpatient clinics that specialize in preventing diabetic foot ulcers has risen along with the increase in the number of patients with diabetes in Japan. However, only a few Japanese reports are available that describe investigations into the long-term occurrence of diabetic foot ulcer and its risk factors [3]. Although diabetic neuropathy and angiopathy have been reported as classical risk factors for diabetic foot ulcer [1, 4], few studies' findings demonstrate the association between the occurrence of diabetic foot ulcer and microangiopathy complications, including albuminuria (Alb) and diabetic retinopathy (DR). Indeed, patients with advanced DR or Alb are very likely to develop diabetic neuropathy or angiopathy. Moreover, DR reduces visual acuity in adults, and the findings from previous studies have shown that a reduction in visual acuity is a risk factor for diabetic foot ulcer [4, 5]. In addition, Alb and elevated serum creatinine (sCr) levels have been reported as risk factors for diabetic foot ulcer [6, 7].

To determine the diabetic foot ulcer incidence and examine the association between diabetic foot ulcer and microangiopathy complications, namely, DR and Alb, we conducted a retrospective study of 1,305 patients with type 2 diabetes who had received in-hospital foot care education.

## 2. Materials and Methods

### 2.1. Study Design and Patients

This retrospective cohort study was conducted at Saiseikai Central Hospital. All subjects who had undergone a 2-week education program for diabetic patients between January 1999 and May 2005 were considered for inclusion in the study. During hospitalization, all subjects attended a 1-hour group lecture about foot care. The following exclusion criteria were applied: (1) the presence of an active diabetic foot ulcer at the time of inclusion, (2) an estimated glomerular filtration rate (eGFR) < 30 mL/min/1.73 m^2^ or a strong suspicion of renal disease other than diabetic nephropathy, for example, glomerulonephritis, and (3) a follow-up period < 1 year. Of the 1,958 study candidates, 648 were transferred to other facilities for medical or personal reasons, or they withdrew from the study for unknown reasons within 1 year, 3 subjects had eGFRs < 30 mL/min/1.73 m^2^, and 2 subjects had active diabetic foot ulcers. Consequently, 1,305 subjects were included in the cohort. Data relating to the patients' ages, sexes, body mass indexes, diabetes durations, and smoking statuses were collected from the medical records at the time of hospitalization. Past histories of cardiovascular disease, including the occurrences of myocardial infarctions, coronary interventions, and ischemic strokes, and the presence of diabetic neuropathy and angiopathy were determined from the patients' medical records.

### 2.2. Clinical Measurements and Assessments

Blood samples were collected in the early morning of day 2 of hospitalization following an overnight fast. The glycosylated hemoglobin (HbA1c) (%; NGSP) levels on admission were determined using high-performance liquid chromatography.

The presence of diabetic neuropathy was evaluated using the Semmes-Weinstein monofilament test and the vibratory sensation test [4]. The Semmes-Weinstein monofilament test involved assessing a foot's sensation using a 10 g monofilament at five sites on the plantar aspect of each foot, namely, the hallux, and the first, second, third, and fifth metatarsal heads [8]. The results from the vibratory sensation test were considered abnormal if the perception of vibration ceased within 10 s after a 128-Hz tuning fork was applied to the medial malleolus. If the sensations from the 10-g monofilament or the vibration sensation tests were reduced, the patient was diagnosed as having sensory neuropathy.

When the patients had histories of intermittent claudication and rest pain, peripheral arterial disease, or absent or reduced pedal pulses, ankle brachial index (ABI) measurements were performed. Every patient rested in a supine position for at least 5 min in a quiet room before the ABI was measured. If either or both ABI values were ≤0.9, the patient was diagnosed with angiopathy.

In addition, 24-hour urine samples were collected from the patients from day 2 until day 3 of hospitalization. The clinical staging of diabetic nephropathy was based mainly on the urinary albumin excretion rate (AER) and, at the advanced stage, it was based on reductions in the eGFR. Alb was defined as a urinary AER >20 *μ*g/min that was determined during the 24-hour urine sample collection. The eGFR, which was used as an index of nephropathy, was obtained using the following formula [9]:

(1) $$\begin{array}{c} eGFR=194\times {sCr}^{-1.094}\times {age}^{-0.287}\times \left(\;woman\;\right)0.739. \end{array}$$

All of the subjects underwent funduscopic examinations that were carried out by a qualified ophthalmologist during or just before hospital admission. A diagnosis of DR was made based on the Davis classification.

The subjects were categorized into four groups based on their diabetic microangiopathy complications at baseline, as follows: Category 1, normoalbuminuria without DR (*n* = 712); Category 2, Alb without DR (*n* = 195); Category 3, normoalbuminuria with DR (*n* = 185); and Category 4, Alb with DR (*n* = 213).

The subjects were treated for diabetes mellitus and, when necessary, for coexisting diseases at our outpatient clinics at intervals of between 1 and 4 months, depending on the status of each disease. The follow-up histories for incident diabetic foot ulcers were obtained from the medical records. The data used in the present study were collected by May 1, 2015.

### 2.3. Ethical Considerations

The study's protocol abided by the Japanese government's ethical guidelines for epidemiological research, and it was reviewed and approved by the ethics committee of Saiseikai Central Hospital (Protocol number 311). All of the procedures were undertaken in accordance with the ethical standards established by the institutional and national committees on human experimentation and in accordance with the Declaration of Helsinki.

### 2.4. Statistical Analyses

The patients' baseline characteristics were determined after they had been stratified into the four categories. The differences among the categories were evaluated using a one-way analysis of variance. The diabetic foot ulcer incidence was calculated in person-years. The 5-year and 10-year cumulative diabetic foot ulcer incidence rates were estimated using the Kaplan-Meier method. The log-rank test was used to examine the differences among the four patient categories in relation to the diabetic foot ulcer incidence. We constructed Cox proportional hazard models to estimate the associations between diabetic complications and the diabetic foot ulcer incidence, using the follow-up time as a dependent variable. Age-, sex-, and multivariable-adjusted hazard ratios (HRs) were calculated using Cox proportional hazards regression models. Model 1 was the age- and sex-adjusted model. The multivariable-adjusted models were adjusted for the presence of diabetic neuropathy alone (Model 2) and for the presence of diabetic neuropathy and angiopathy (Model 3). The multivariable-adjusted HRs were used to calculate the relative risks of diabetic foot ulcer occurrence in each diabetic microangiopathy complications category. A *P* value < 0.05 was considered to indicate statistical significance. STATA version 10 (StataCorp LP, College Station, TX, USA) was used for the statistical calculations.

## 3. Results

### 3.1. Baseline Clinical Characteristics of the Patients in the Four Categories

Table 1 shows the baseline characteristics of the study participants who were grouped according to their diabetic microangiopathy complications. The mean age of the study cohort was approximately 60 years, approximately 70% of the patients were men, and the mean diabetes duration was approximately 10 years. The mean HbA1c level was 9.1%. Of the 1,305 study participants, 54.6% (*n* = 712) were assigned to Category 1, 14.9% (*n* = 195) were assigned to Category 2, 14.2% (*n* = 185) were assigned to Category 3, and 16.3% (*n* = 213) were assigned to Category 4. Several baseline characteristics showed significant differences among the microangiopathy complications categories. For example, the subjects in Category 4 were older, were predominantly male, and they had longer disease durations compared with the subjects in the other categories.

### 3.2. Cox Regression Analysis of Albuminuria and Diabetic Retinopathy at Baseline and the Diabetic Foot Ulcer Incidence

Of the 1,305 subjects evaluated, 93 died, 28 were transferred to other facilities for medical or personal reasons, and 201 withdrew for unknown reasons during the 10-year follow-up period. The follow-up rate at 10 years was 75.3%. During the median follow-up period of 11.8 years, 50 cases were diagnosed with new-onset diabetic foot ulcers, yielding 14,291 person-years (Table 2). The annual diabetic foot ulcer incidence rates were 1.6, 1.5, 3.4, and 12.5 per 1,000 person-years in Categories 1, 2, 3, and 4, respectively.

**Figure 1:**
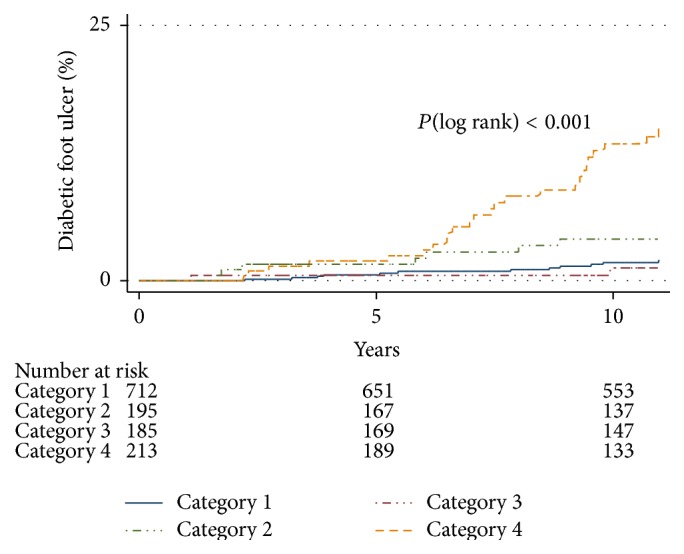
Unadjusted Kaplan-Meier estimates of the occurrence of diabetic foot ulcers over 10 years. The data shown are for patients with different microangiopathy complications at baseline.

The Kaplan-Meier curves showed that the individuals in Category 4 had a significantly higher diabetic foot ulcer incidence (log-rank test, *P* < 0.001) (Figure 1). The 10-year cumulative diabetic foot ulcer incidence increased according to the microangiopathy complications category, with 1.7%, 1.2%, 4.2%, and 12.7% of subjects developing diabetic foot ulcers in Categories 1, 2, 3, and 4, respectively (Table 2).

Associations between the presence of DR, Alb, diabetic neuropathy, and angiopathy and the diabetic foot ulcer incidence were identified using Cox regression analysis (Table 3). After adjusting for age and sex (Model 1), the presence of diabetic microangiopathy complications was positively and significantly associated with the diabetic foot ulcer incidence. In Model 3, which was additionally adjusted for the presence of diabetic neuropathy and angiopathy, the HRs and the 95% confidence intervals (CIs) for the diabetic foot ulcer incidence were 0.66 (95% CI, 0.18–2.36), 1.72 (95% CI, 0.67–4.42), and 3.17 (95% CI 1.52–6.61; *P* = 0.002) for Categories 2, 3, and 4, respectively, compared with Category 1.

## 4. Discussion

This study aimed to determine the diabetic foot ulcer incidence in patients with type 2 diabetes and to investigate the association between diabetic foot ulcer and microangiopathy. Following in-hospital education, the 5-year and 10-year cumulative diabetic foot ulcer incidence rates in patients with type 2 diabetes were 1.9% and 3.7%, respectively. Importantly, the 10-year cumulative diabetic foot ulcer incidence rate in Category 4, which comprised patients with both DR and Alb, was 12.7% and was up to threefold higher than the 10-year cumulative diabetic foot ulcer incidence rate in Category 1. After adjusting for the presence of diabetic neuropathy and angiopathy, the presence of diabetic microangiopathy complications was positively and significantly associated with the occurrence of diabetic foot ulcers. The findings from this study provide information about the risk of patients developing diabetic foot ulcers, and they enable more realistic risk estimations in relation to the occurrence of diabetic foot ulcers in individuals with DR and Alb.

Although the number of foot care outpatient departments has grown in response to the increase in the number of patients with diabetes in Japan, few long-term follow-up studies have investigated the diabetic foot ulcer incidence, and the results from short-term studies only are available in the literature [3]. Reports from Asian countries are particularly uncommon [10], and an association between the diabetic foot ulcer incidence and the presence of DR and/or Alb has not been proposed. Diabetic microangiopathy progresses with the duration of a patient's diabetes, and the diabetic complications observed are interrelated. The results from this study may enable type 2 diabetes patients with DR and/or Alb to understand the actual risk in relation to diabetic foot ulcer development. Moreover, the 10-year cumulative diabetic foot ulcer incidence rates in the patients in Categories 3 and 4, which included those with DR, were higher than the 10-year cumulative diabetic foot ulcer incidence rates in the patients in Categories 1 and 2, which contained patients who did not have DR, indicating that DR is a particularly important factor associated with the occurrence of diabetic foot ulcers. Indeed, Boyko et al. [6] and Leese et al. [11] found that poor vision, which was defined as a corrected visual acuity of 20/40 or lower, is a risk factor for diabetic foot ulcer. In addition, DR is a risk factor for all causes of death, cardiovascular events, and arteriosclerosis. In their study, Ohtomo et al. [12] found that approximately 20% of the patients with DR had coronary artery disease, which may reflect impaired vascular perfusion caused by arteriosclerosis. In another study, Ogawa et al. [13] reported that diabetic neuropathy was present in all of the patients with type 2 diabetes who were hospitalized for diabetic foot ulcers and that DR and nephropathy were present in 96.4% and 78.6% of the patients, respectively. Thus, peripheral arterial disease may be present in patients with early DR. The incidence of DR in Japanese patients with type 2 diabetes is 38.3/1,000 person-years, and the DR progression rate has been reported as 21.1/1,000 person-years [14]. Since subjective symptoms are often absent, even when DR is at an advanced stage, it is important to encourage patients with definitively diagnosed or suspected diabetes to visit an ophthalmology department.

The diabetic foot ulcer incidence has been investigated previously. From their study, Peters and Lavery [15] reported that during the 3-year follow-up period, foot ulceration occurred in 14.3%, 18.8%, and 55.8% of the patients with diabetic neuropathy, diabetic neuropathy combined with foot deformities or peripheral arterial disease, and previous histories of foot ulceration, respectively. Moss et al. [16] followed up with 906 patients who were diagnosed with type 2 diabetes before they were 30 years old and 984 patients who were diagnosed with type 2 diabetes after they were 30 years old for 14 years, and they found that amputations caused by diabetic foot ulcers were required in 7.2% and 9.9% of these patients, respectively, and they concluded that severe retinopathy was a risk factor for amputation. In our study cohort, the diabetic foot ulcer frequency was lower than the frequencies reported from these aforementioned studies, which could be a consequence of the in-hospital education that our patients received. In a Japanese study, Oe et al. [3] followed up with 578 patients with diabetes who visited a specialized outpatient department for 5 years, and they found diabetic foot ulcers in 6 (1.2%) patients. However, 70% of the subjects did not have diabetic neuropathy or angiopathy [3]; therefore, the patients' backgrounds differed from those in the current study, in which 40% of the patients had diabetic neuropathy.

Despite the patients in the current study attending a group lecture about foot care as part of an in-hospital education program, the patients with type 2 diabetes, DR, and Alb developed diabetic foot ulcers, and a sharp increase in the cumulative diabetic foot ulcer incidence was observed after 5 years (Figure 1). Therefore, since the complications associated with diabetes tend to progress with its duration, patients with microangiopathy may require recurring outpatient education programs.

Several limitations should be considered when interpreting the results from the present study. First, this was a hospital-based cohort study; hence, a selection bias cannot be excluded. Second, a considerable percentage of the subjects were lost to follow-up during the study period. In addition, changes in the glucose levels and changes to the therapeutic regimens were not considered during the outpatient treatment period. Furthermore, we did not identify an association between DR and visual acuity. Finally, the impacts of the foot care and education programs on the study's results are unknown. Future investigations into the occurrence of diabetic foot ulcer in Japan should account for these limitations.

## 5. Conclusions

The findings from this study showed that diabetic individuals with DR and Alb were at a significantly increased risk of developing diabetic foot ulcers. From the perspective of a diabetic patient's evaluation of risk, the presence of asymptomatic DR and/or Alb may not be considered serious; hence, early intervention and long-term follow-up programs are crucial to prevent diabetic foot ulcers.

## Figures and Tables

**Table 1:** Baseline characteristics of the study population according to the microangiopathy complications category.

	All patients	Category 1: NA (DR−)	Category 2: Alb (DR−)	Category 3: NA (DR+)	Category 4: Alb (DR+)	*P* values
Number of patients, *n* (number of men, *n*)	1,305 (904)	712 (472)	195 (156)	185 (112)	213 (164)	
Age (years)	59.9 ± 9.1	59.4 ± 8.9	60.1 ± 9.3	61.4 ± 8.8	60.3 ± 9.4	0.03
BMI (kg/m^2^)	23.8 ± 3.9	23.4 ± 3.6	25.5 ± 4.7	22.8 ± 3.4	24.3 ± 4.1	<0.001
Diabetes mellitus duration (years)	10.3 ± 8.2	8.4 ± 7.3	8.5 ± 7.9	14.3 ± 8.0	15.1 ± 8.6	<0.001
HbA1c (%; NGSP)	9.2 ± 1.8	9.1 ± 1.8	9.2 ± 1.8	9.1 ± 1.5	9.5 ± 1.8	0.0092
Neuropathy, *n* (%)	520 (39.8)	202 (28.4)	67 (34.4)	99 (53.5)	152 (71.4)	<0.001
Angiopathy, *n* (%)	33 (2.5)	11 (1.5)	9 (4.6)	2 (1.1)	11 (5.2)	0.003
Prior cardiovascular disease, *n* (%)	136 (10.4)	49 (6.9)	29 (14.9)	25 (13.5)	33 (15.5)	<0.001
Smoker, *n*, none/past/current	707/276/322	388/153/171	86/53/56	118/30/37	115/40/58	0.011
Treated with insulin, *n* (%)	528 (40.5)	211 (29.6)	66 (33.9)	111 (60.0)	140 (65.7)	<0.001
Diabetic retinopathy, *n*,	907/259/139	712/0/0	195/0/0	0/125/60	0/134/79	<0.001
none/background/more advanced stage or prior photocoagulation						
eGFR (mL/min/1.73 m^2^)	79.3 ± 20.1	80.8 ± 18.3	77.8 ± 22.4	78.9 ± 20.6	75.7 ± 22.5	0.02
Diabetic nephropathy, *n*, none/microalbuminuria/macroalbuminuria	897/327/81	712/0/0	0/173/22	185/0/0	0/154/59	<0.001

BMI: body mass index; HbA1: glycated hemoglobin; NGSP: National Glycohemoglobin Standardization Program; eGFR: estimated glomerular filtration rate; NR: normoalbuminuria; Alb: albuminuria; DR: diabetic retinopathy.

Data are expressed as the number of patients (%), number of patients, or the means ± standard deviations.

**Table 2:** Diabetic foot ulcer incidence according to the microangiopathy complications category at baseline.

Category	*n*	Number of cases, *n*	Time at risk (person-years)	Incidence (per 1,000 person-years)	5-year cumulative incidence (%)	(95% CI)	10-year cumulative incidence (%)	(95% CI)
Category 1	712	13	8,063	1.6	1.2	(0.4–3.1)	1.7	(0.9–3.1)
Category 2	195	3	2,025	1.5	1.1	(0.2–7.6)	1.2	(0.3–4.8)
Category 3	185	7	2,054	3.4	3.4	(1.1–10.4)	4.2	(2.0–8.8)
Category 4	213	27	2,148	12.5	3.9	(1.4–10.4)	12.7	(8.4–19.1)
Total	1,305	50	14,291	3.5	1.9	(1.1–3.3)	3.7	(2.7–5.0)

CI: confidence interval.

**Table 3:** Hazard ratios for diabetic foot ulcer in the adjusted models according to the microangiopathy complications category at baseline.

	Model 1	*P* values	Model 2	*P* values	Model 3	*P* values
	HR (95% CI)		Adjusted HR (95% CI)		Adjusted HR (95% CI)	
Category 1	1.00		1.00		1.00	
Category 2	0.74 (0.21–2.64)	0.64	0.73 (0.21–2.59)	0.62	0.66 (0.18–2.36)	0.52
Category 3	2.26 (0.91–5.69)	0.08	1.60 (0.63–4.07)	0.32	1.72 (0.67–4.42)	0.25
Category 4	6.84 (3.48–13.41)	<0.001	3.91 (1.91–7.95)	<0.001	3.17 (1.52–6.61)	0.002
Neuropathy	—	—	4.37 (2.03–9.43)	<0.001	3.67 (1.66–8.01)	0.001
Angiopathy	—	—	—		7.74 (3.87–15.5)	<0.001

Model 1: adjusted for age and sex; Model 2: Model 1 + adjusted for neuropathy; Model 3: Model 2 + adjusted for angiopathy.

CI: confidence interval; HR: hazard ratio.
